# PKC Activation in Niemann Pick C1 Cells Restores Subcellular Cholesterol Transport

**DOI:** 10.1371/journal.pone.0074169

**Published:** 2013-08-15

**Authors:** Farshad Tamari, Fannie W. Chen, Chunlei Li, Jagrutiben Chaudhari, Yiannis A. Ioannou

**Affiliations:** 1 Department of Genetics and Genomic Sciences, the Mount Sinai School of Medicine, New York, New York, United States of America; 2 Department of Biological Sciences, Kingsborough Community College, Brooklyn, New York, United States of America; International Centre for Genetic Engineering and Biotechnology, Italy

## Abstract

Activation of protein kinase C (PKC) has previously been shown to ameliorate the cholesterol transport defect in Niemann Pick Type C1 (NPC1) cells, presumably by increasing the soluble levels of one of its substrates, vimentin. This activity would then restore the vimentin cycle in these cells and allow vimentin-dependent retrograde transport to proceed. Here, we further investigate the effects of PKC activation in NPC1 cells by evaluating different isoforms for their ability to solubilize vimentin and correct the NPC1 cholesterol storage phenotype. We also examine the effects of PKC activators, including free fatty acids and the PKC-specific activator diazoxide, on the NPC1 disease phenotype. Our results indicate that PKC isoforms α, βII, and ε have the greatest effects on vimentin solubilization. Furthermore, expression or activation of PKCε in NPC1 cells dramatically reduces the amount of stored cholesterol and restores cholesterol transport out of endocytic vesicles. These results provide further support for the contribution of PKCs in NPC1 disease pathogenesis and suggest that PKCs may be targeted in future efforts to develop therapeutics for NPC1 disease.

## Introduction

The protein kinase C (PKC) family of enzymes is responsible for a multitude of cellular processes through the enzymes’ ability to regulate proteins via signal transduction cascades [1]. The members of this kinase family are structurally and functionally similar [2] and are categorized into conventional (α, βI, βII and γ), novel (δ, ε, η, and θ), and atypical isoforms (ζ and λ) [3]. These isoforms have been implicated in a variety of diseases and pathological conditions over the years [4].

A previously unappreciated role for PKCs in Niemann-Pick Type C (NPC1) disease was revealed by our observations that the intermediate filament vimentin is hypophosphorylated in NPC1 cells compared to Wt cells and that this hypophosphorylation results from reduced PKC activity [5]. Vimentin is involved in a variety of cellular processes, including vesicular membrane transport [6,7], signal transduction [8,9] and cell motility [10]. Similar to NPC1 cells, cells lacking vimentin are unable to transport LDL-derived cholesterol from their lysosomes to the endoplasmic reticulum for esterification [11]. The decreased vimentin phosphorylation in NPC1 cells reduces the pool of soluble vimentin, likely disrupting the vimentin cycle, which is necessary for transport to take place [12,13]. Vimentin has been shown to be phosphorylated by several proteins, including the PKCs [14] and in particular the α [15], ε [10] and βII [16,17] isoforms.

In these studies we investigate the differences between WT and NPC1 cells with respect to their levels of soluble vimentin and evaluate the ability of the different PKC isoforms to solubilize vimentin in NPC1 cells. We find that the PKC α, βII, and ε isoforms can ameliorate the NPC1 cholesterol transport block as determined by esterification assays and filipin staining. Furthermore, fatty acid activators of PKCs have a similar and additive effect, suggesting that specific PKC isoforms could be therapeutically targeted for treatment of this disease.

## Results

### PKC Expression Increases the Levels of Soluble Vimentin in NPC1 Cells

We have previously shown that NPC1 cells with missense or null (NPC1_o_) mutations contain decreased or virtually undetectable levels of soluble phosphorylated vimentin relative to Wt cells, respectively [5]. Furthermore, the vimentin present in NPC1 cells exists as large disorganized filaments (dephosphorylated state) near the plasma membrane. Thus, NPC1 cells behave essentially as vimentin-null cells, which, similar to NPC1 cells, are unable to esterify LDL-derived cholesterol [11]. In extending those studies, we hypothesized that decreased vimentin phosphorylation was the result of protein kinase C (PKC) inhibition in NPC1 cells. In support of this, we observed in that study that treatment of NPC cells with the PKC activator phorbol-12-myristate-13-acetate (PMA) increased levels of soluble vimentin and ameliorated the NPC lipid storage phenotype, whereas conversely, treatment of WT cells with PKC inhibitors resulted in the disappearance of soluble vimentin in those cells. These results strongly implicate PKC in the maintenance of the soluble vimentin pool in cells and by extension normal lysosomal cholesterol efflux. Here we extend those studies by evaluating different PKC isoforms and their effects on soluble vimentin levels in NPC cells.

**Figure 1:**
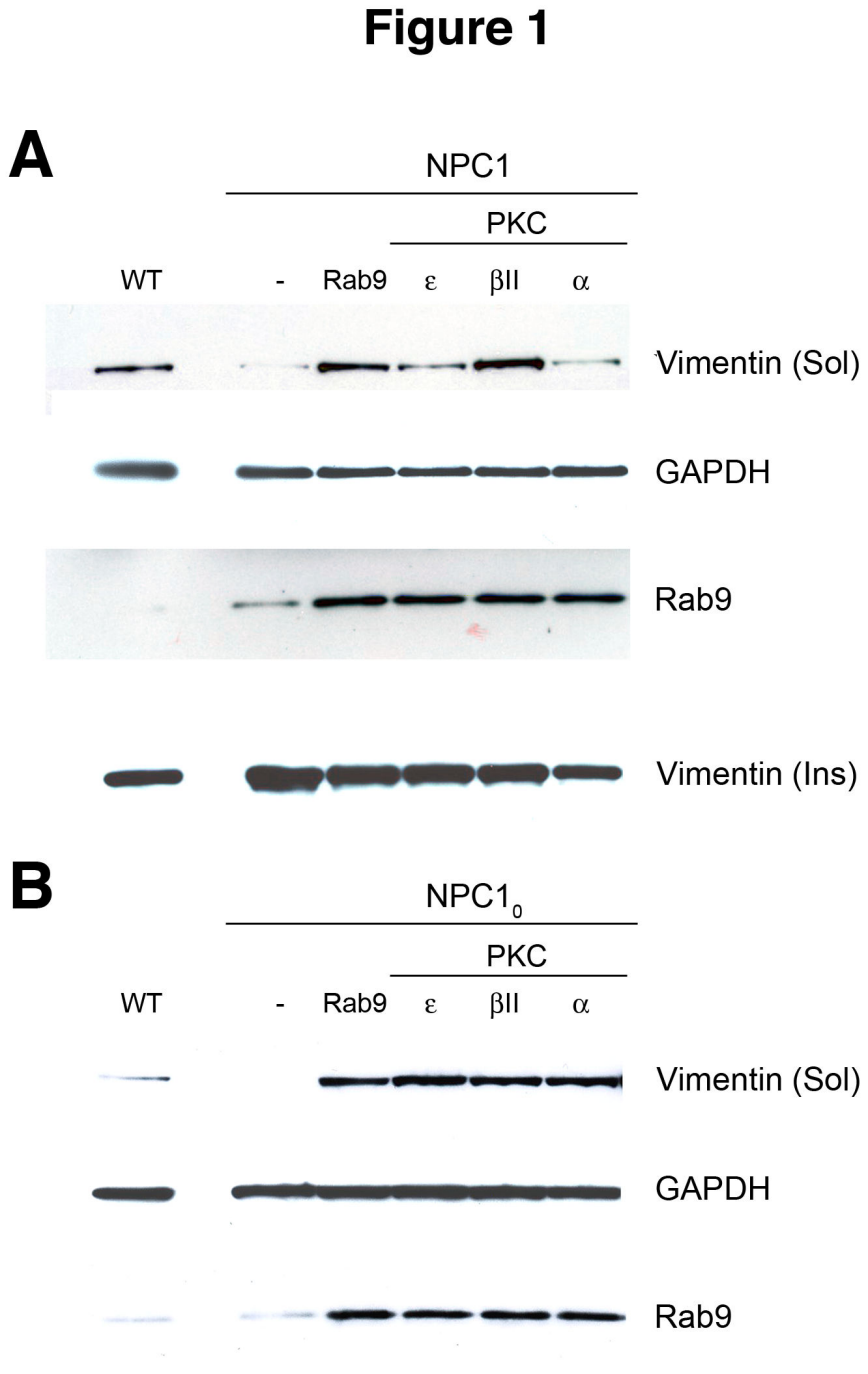
Effects of transient PKC expression on vimentin solubilization in human NPC1 cells. Representative western blot analyses of soluble and insoluble vimentin levels in human NPC1 3123 (A) and human nullNPC1_0_ (B) cells transfected with PKC ε, β, or α show that the three isoforms increase levels of soluble vimentin and Rab9 with a concurrent decrease of insoluble vimentin relative to untransfected cells (-). The levels of vimentin solubilized are similar to that seen in cells expressing Rab9 (Rab9). The blots shown are representative of at least 3 independent experiments.

The PKC isoforms α, βII, and ε have been implicated in vimentin phosphorylation [10,17,18]; therefore, we first focused our studies on these isoforms. They were transiently expressed in human NPC1 cells and their effects on soluble vimentin levels were characterized. Expression of PKC βII caused a significant increase in soluble vimentin levels (~38-fold higher than untransfected NPC1 cells), which was higher than the levels seen in Wt cells (~20-fold higher than NPC1 cells), whereas expression of PKCs α or ε caused smaller but still significant increases (~3-fold and ~7-fold, respectively) in soluble vimentin levels (Figure 1). As a control, expression of Rab9 in these cells also led to a significant increase (~30-fold) in soluble vimentin, consistent with what we previously reported [5]. Interestingly, all three isoforms resulted in an increase of soluble Rab9 levels to a similar degree (~2500-fold higher than untransfected NPC1 cells). Furthermore, insoluble vimentin levels decreased as soluble vimentin levels increased in PKC-expressing cells, suggesting that the increase in soluble vimentin was due to solubilization (phosphorylation) of insoluble vimentin (Figure 1A).

Similarly, in the severely affected NPC1_0_ cells, which normally have almost no detectable soluble vimentin, expression of any of the three PKC isoforms resulted in increased levels of soluble vimentin (Figure 1B). With respect to vimentin solubilization, all three isoforms work equally well in the NPC1_0_ cells, in contrast to the NPC1 cells, in which the βII isoform seemed to be the most effective in solubilizing vimentin. Furthermore, soluble Rab9 levels also increased to similar levels as a result of PKC expression (Figure 1B), a result also seen in PKC-expressing NPC1 cells (Figure 1A).

### PKC Expression Induces Rab9 Dissociation from Vimentin

**Figure 2:**
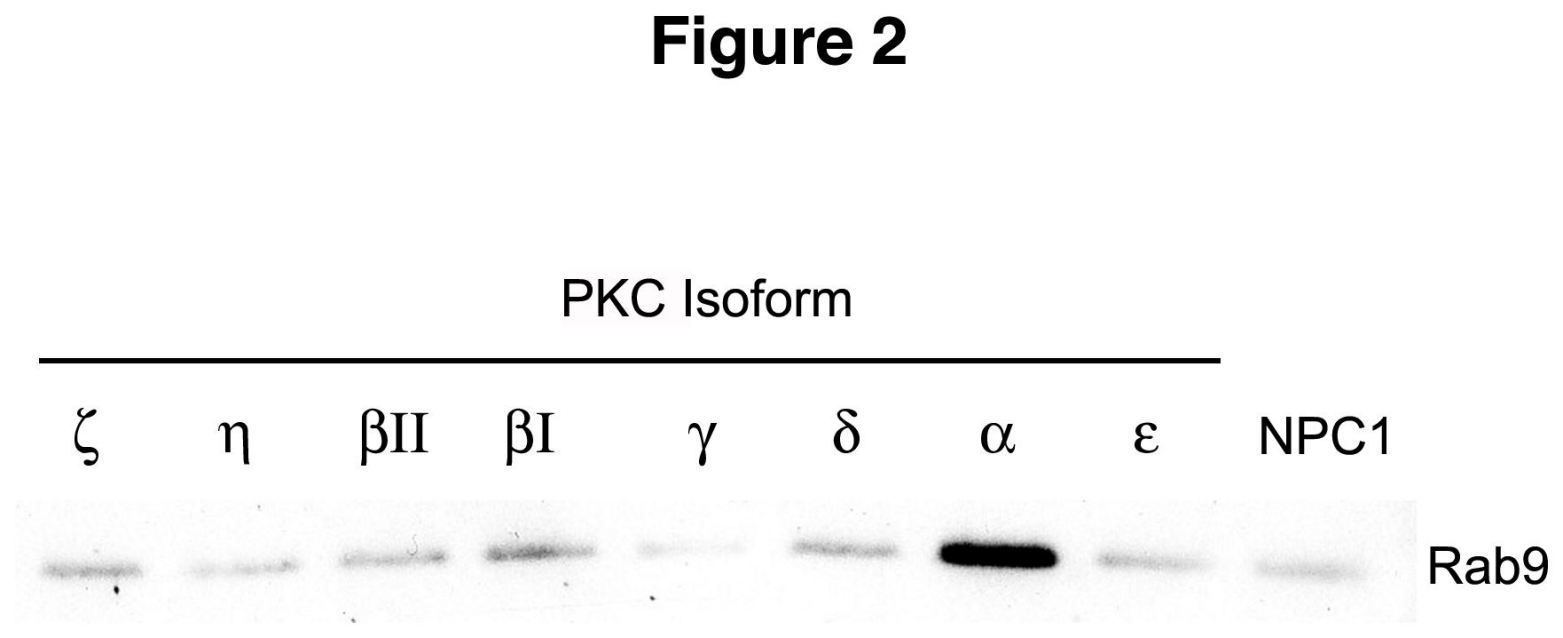
Rab9 release from insoluble vimentin fraction of NPC1 cell lysates. The insoluble vimentin fraction from NPC1 cell lysates was incubated with various PKC isoforms. All isoforms tested can effect Rab9 release to some degree from the insoluble vimentin fraction, with PKCα being the most effective and PKCγ being the least effective. The blots shown are representative of at least 3 independent experiments.

The observations that the small GTPase Rab9 is trapped in vimentin filaments in NPC1 cells [5] and that Rab9 overexpression corrects the NPC1 phenotype [19] strongly suggest that Rab9 availability is reduced in NPC1 cells. The disease cells may attempt to compensate for this deficit by upregulating Rab9 protein expression. This idea is supported by the fact that NPC1 cells do contain more Rab9 protein than Wt cells (Figure 1A and [19]). As described above, PKC expression increased not only soluble vimentin levels but also soluble Rab9 levels significantly (Figure 1A and 1B). To determine whether the increased Rab9 levels in PKC-expressing cells was a result of Rab9 release from insoluble vimentin after it was phosphorylated, we performed PKC assays *in vitro* with nine purified PKC isoforms using the insoluble vimentin fraction of NPC1 cell lysates as the PKC substrate. All isoforms were able to effect Rab9 release from insoluble vimentin to varying degrees (Figure 2), suggesting that, at least *in vitro*, most PKC isoforms can catalyze vimentin phosphorylation and Rab9 release. Interestingly, PKC α caused the greatest increase in Rab9 release, while PKC βII and ε were less effective and PKC γ was almost ineffective. These results differ from the results of transiently transfected PKC-expressing cells, which suggest that PKC βII is more effective in increasing soluble vimentin levels than PKC α and ε (Figure 1A). The discrepancies in isoform effectiveness may be due to the nature of the assays, the inherent activity of each isoform, or the *in vivo* subcellular location of the different isoforms and their access to vimentin [10,16,17,20]; however, it is clear that PKC is able to solubilize vimentin and in doing so release entrapped Rab9.

### Overexpression of PKCs Induces a Partial Correction of the NPC1 Phenotype

**Figure 3:**
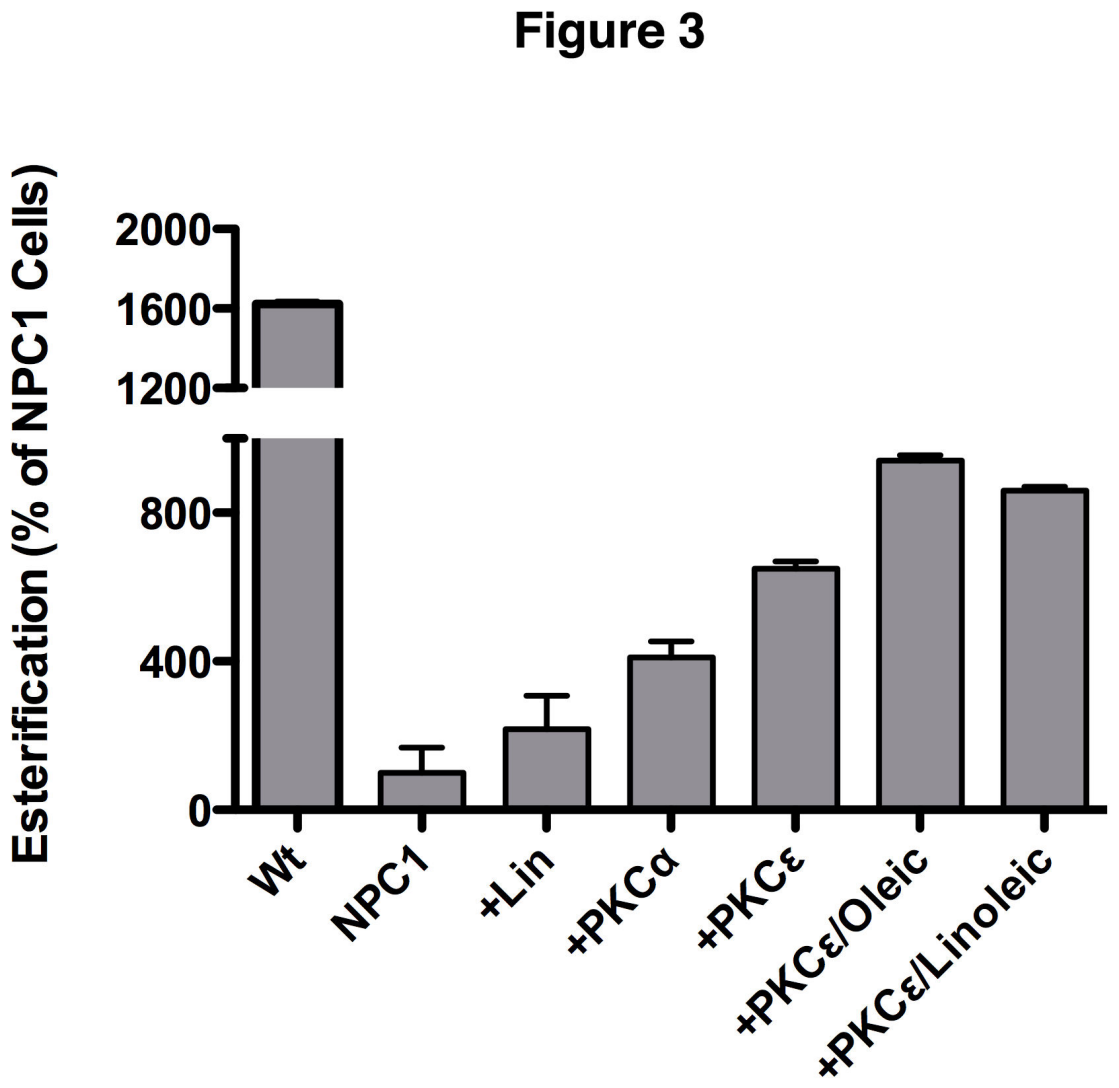
Effects of PKCs and fatty acids on cholesterol esterification in M12 NPC1 CHO cells. M12 cells were treated with 50µg/ml fatty acids for 2 days and then transfected with the indicated PKC isoforms. Following transfection, cholesterol transport was assessed by esterification assay. Both free fatty acids and PKCs alleviate the cholesterol transport defect of NPC1 cells and their effects appear to be additive.

Based on the data above and observations that Rab9 overexpression results in increased soluble vimentin and correction of the NPC1 phenotype [5], we next determined whether increased vimentin solubility caused by PKC overexpression would also result in correction of the NPC1 phenotype. NPC1 CHO (M12) cells containing a deletion of the NPC1 locus [21] were transfected with PKC α or PKC ε and the amount of LDL-derived free cholesterol transported from the E/L system to the ER for esterification by acyl-CoA: cholesterol acyltransferase (ACAT) [22] was measured. As expected, esterification levels for M12 cells were less than 10% of the esterification activity of the parental Wt CHO cells (Figure 3), which is consistent with a block in cholesterol transport out of the E/L system. Expression of PKC α or PKC ε ameliorated the cholesterol transport block, increasing the level of M12 cell esterification by approximately 4- and 6.5-fold, respectively, over that of untransfected M12 cells (Figure 3, +PKCα, +PKCε). These results indicate that solubilization of vimentin mediated by expression of PKCs can partially release the NPC1 lipid transport block.

**Figure 4:**
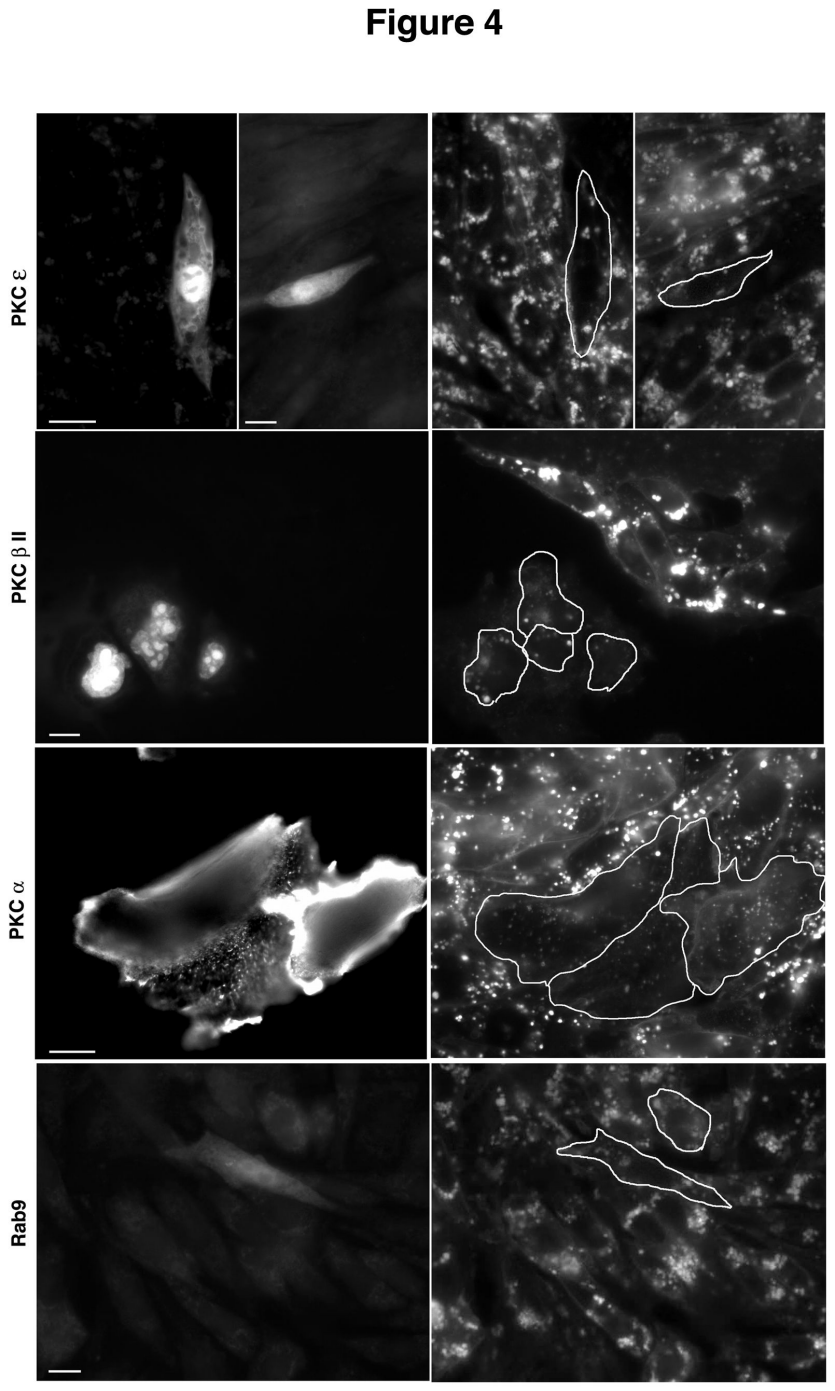
Effects of transient PKC expression on the NPC1 phenotype. M12 cells were transfected with PKC isoforms or Rab9 for 48 hrs and then analyzed by filipin staining for cholesterol storage. Cells positive for transfection stain positive for GFP (left panel) and show decreased filipin staining (outlined cells, right panel) compared to surrounding untransfected cells, confirming the role of PKCs in mobilizing stored cholesterol from the NPC1 endosomes. Bar, 20μ.

Cholesterol storage in PKC-transfected cells was also determined qualitatively by staining with filipin, a fluorescent probe that binds to free cholesterol [23]. This analysis yielded similar results to the esterification studies shown in Figure 3. Cells that were positive (as determined by GFP co-expression) for PKCα, PKCε, or PKCβII showed significantly less filipin staining than surrounding untransfected cells (Figure 4). These results were similar to those seen in cells over-expressing Rab9 (Figure 4, Rab9), which has previously been shown to correct the NPC1 cholesterol storage phenotype [19].

### Exposure to Fatty Acids Increases Soluble Vimentin Levels in NPC1 Cells

Fatty acids and in particular oleic acid have been shown to induce PKC activity [24], whereas a downstream metabolite of linoleic acid, DCP-LA (8-[2-(2-pentyl-cyclopropylmethyl)-cyclopropyl]-octanoic acid), has been shown to potently activate PKC ε [25,26]. Furthermore, we have shown previously that NPC1 endosomes store large amounts of fatty acids [27], which could potentially limit the amount of free fatty acids available to the cell for PKC activation and other processes.

**Figure 5:**
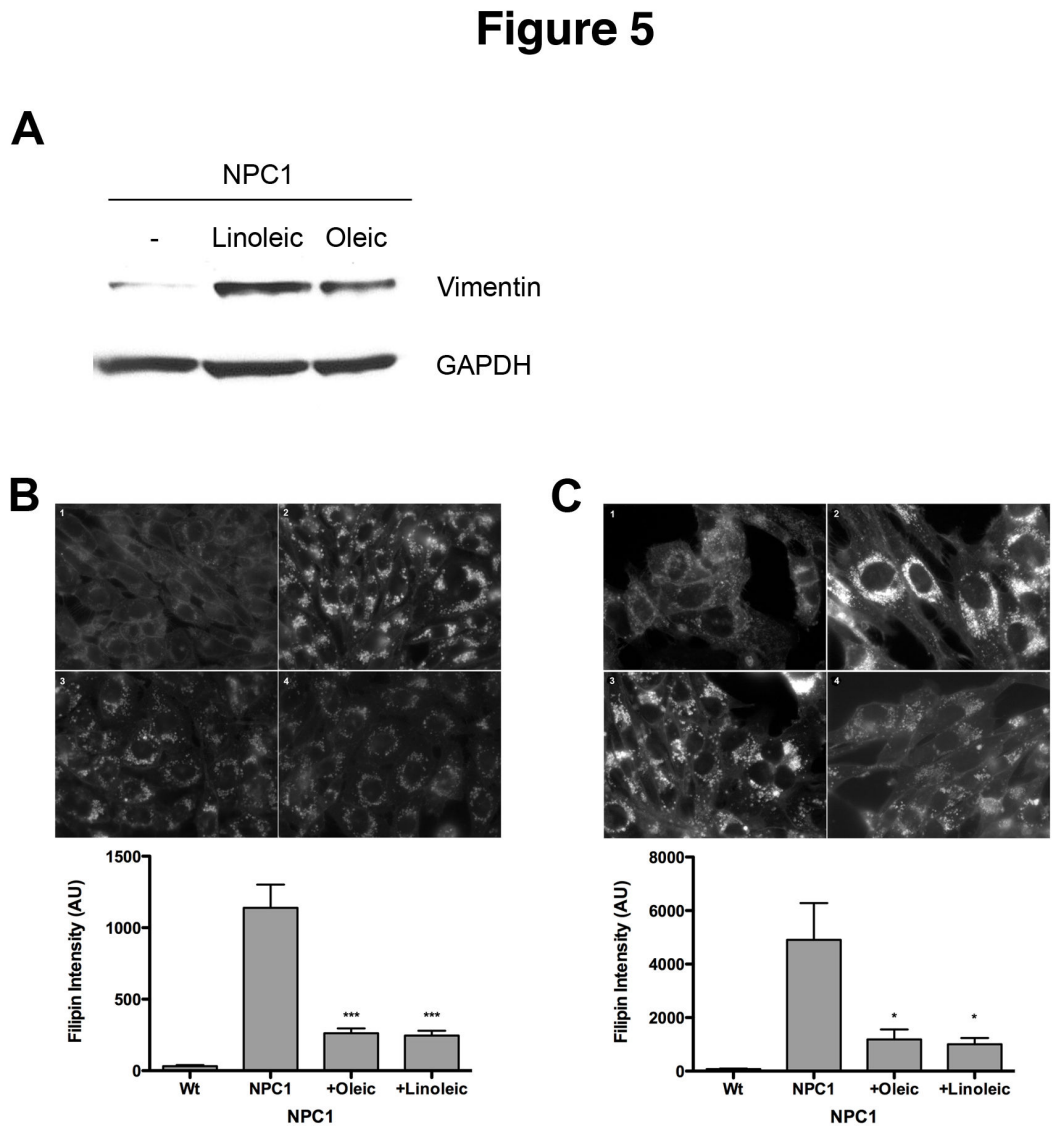
Effects of fatty acids on vimentin solubilization and the NPC1 phenotype. (A) Human NPC1 3123 cells were treated with 50µg/ml linoleic or oleic acid for 24 hrs, after which the levels of soluble vimentin were analyzed by Western blotting. Cholesterol storage in NPC1 CHO (B) or human 3123 (C) cells was analyzed by filipin staining. Fluorescence intensity was quantitated in at least 150 cells for each sample. The bar graph represents average values ±SEM from 3 independent experiments. * and *** denote statistically significant differences between treated and untreated cells with P<0.05 and P<0.0001, respectively, as determined by Student’s t-test.

To determine if exogenously added fatty acids can increase vimentin solubilization in NPC1 cells, human NPC1 fibroblasts were treated with oleic or linoleic acid for 48 hours and the levels of soluble vimentin in cell lysates were analyzed. As expected, NPC1 fibroblasts contain very little soluble vimentin (Figure 5A and [5]). Treatment with either oleic or linoleic acid significantly increases the amount of soluble vimentin, with oleic acid being slightly more effective. These results suggest that exogenously added fatty acids can effect vimentin solubilization in NPC1 cells, presumably by activating PKCs.

### Exposure to Fatty Acids Induces Correction of the NPC1 Phenotype

Since fatty acids increase solubilization of vimentin in NPC1 cells, it seemed reasonable that they might also improve the NPC1 phenotype. M12 CHO cells (Figure 5B) and human NPC1 fibroblasts (Figure 5C) were treated with each fatty acid and then stained with filipin. As expected, Wt cells (Figures 5B/C, 1) stain very weakly with filipin, whereas NPC1 (Figures 5B/C, 2) cells contain bright, punctate staining that is indicative of free cholesterol in endocytic vesicles. Filipin fluorescence in NPC1 cells from both species was significantly decreased after exposure to either fatty acid (Figures 5B/C, 3 and 4). Following quantitation of filipin fluorescence levels by integrated morphometry, both fatty acids were found to dramatically reduce the levels of cholesterol accumulation in both NPC1 cell lines, reducing filipin fluorescence to ~75% of levels in untreated cells (Figure 5B/C, graphs). Human fibroblasts exhibit more heterogeneous filipin staining patterns than either CHO or mouse NPC1 cell lines, which is reflected in the higher standard deviation in untreated 3123 cells (Figure 5C, graph).

The effect of fatty acids on cholesterol esterification in M12 cells was also evaluated. Linoleic acid increased cholesterol esterification by greater than 2-fold compared to untreated M12 cells (Figure 3, +Lin). In M12 cells treated with oleic or linoleic acids followed by expression of PKC ε, the correction of the NPC1 phenotype was more pronounced, with esterification levels that were ~3.5-fold over untreated cells (Figure 3, +PKCε/oleic and +PKCε/linoleic). These results suggest that fatty acids and PKC expression have an additive effect on correction of the NPC1 cholesterol transport block.

**Figure 6:**
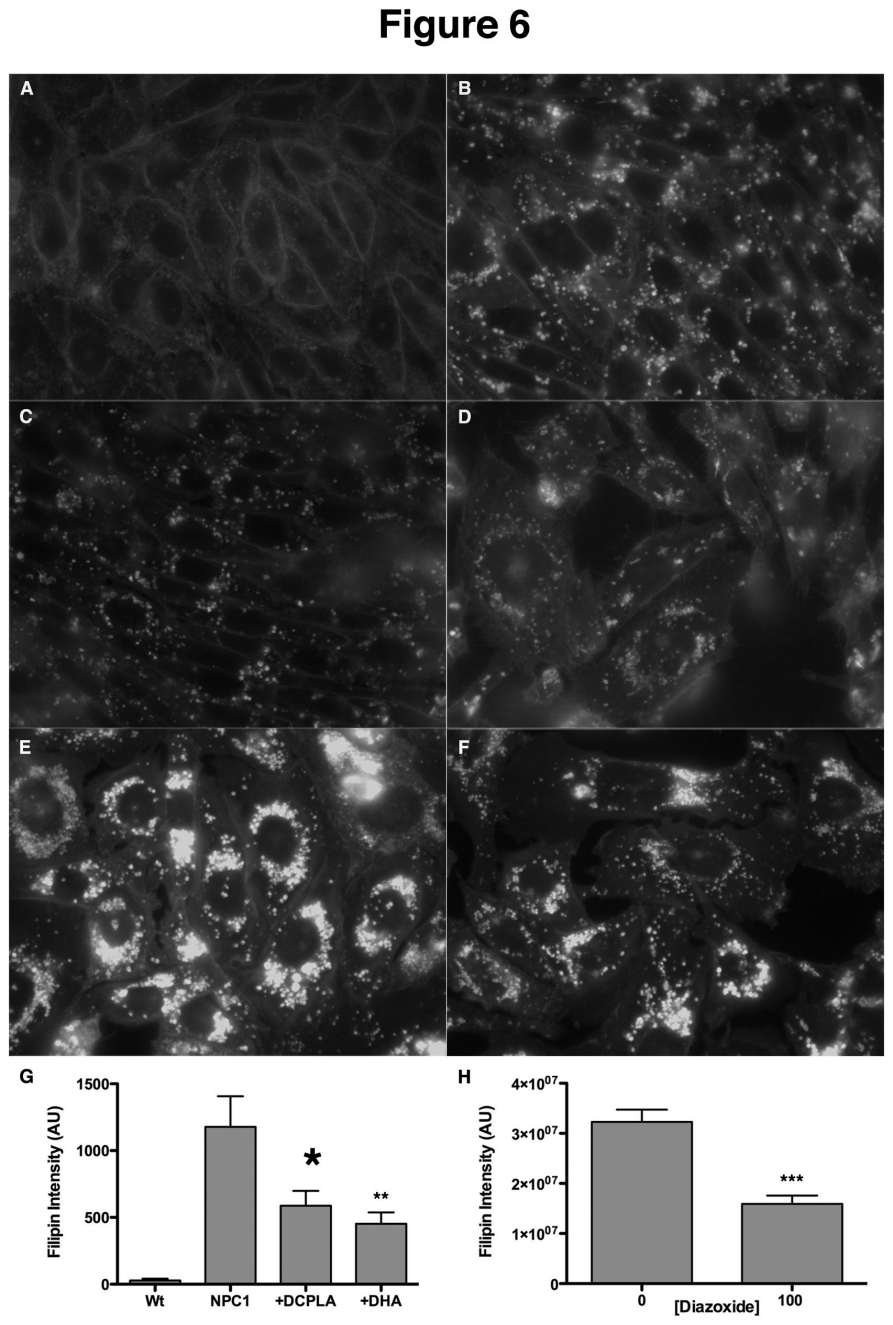
Effects of PKC activation on the NPC1 phenotype. NPC1 CHO cells (B and E) were treated with 100µM DCP-LA (C), 10µM DHA (D), or 100µM diazoxide (E) and cholesterol storage was quantified by filipin fluorescence. (A) Wt CHO cells. Filipin intensity was quantitated in at least 150 cells for each sample. The bar graph represents average values ±SEM from 3 independent experiments. *, **, and *** denote statistically significant differences between treated and untreated cells with P<0.05, P<0.01 and P<0.0001, respectively, as determined by Student’s t-test.

To further characterize the effects of fatty acids on the NPC phenotype, we also tested the ability of docosahexanoic acid (DHA) and a metabolite of linoleic acid, DCP-LA, for their ability to decrease cholesterol storage in M12 CHO cells. Both fatty acids have been shown to potently activate PKC ε [24,28]. Treatment of M12 CHO cells with these compounds overnight resulted in decreased cholesterol storage (Figure 6C/D) relative to untreated cells (Figure 6B). DCP-LA (Figure 6C) was slightly more effective than DHA (Figure 6D). Quantitation of filipin intensity in these cells revealed that both compounds reduced cholesterol storage in M12 CHO cells by ~50% (Figure 6G), lending further support to the positive effect of free fatty acids on the NPC disease phenotype, possibly through activation of PKC ε.

To provide further support for the role of PKCs in NPC rescue, M12 cells were treated with diazoxide, which has been shown to activate PKC ε [29]. This treatment resulted in reduced cholesterol accumulation in M12 CHO cells (Figure 6F) relative to untreated cells (Figure 6E). Quantitation of the filipin intensity in these cells indicated that diazoxide reduced cholesterol storage by ~50%, similar to the results seen with the free fatty acids (Figure 6G).

**Figure 7:**
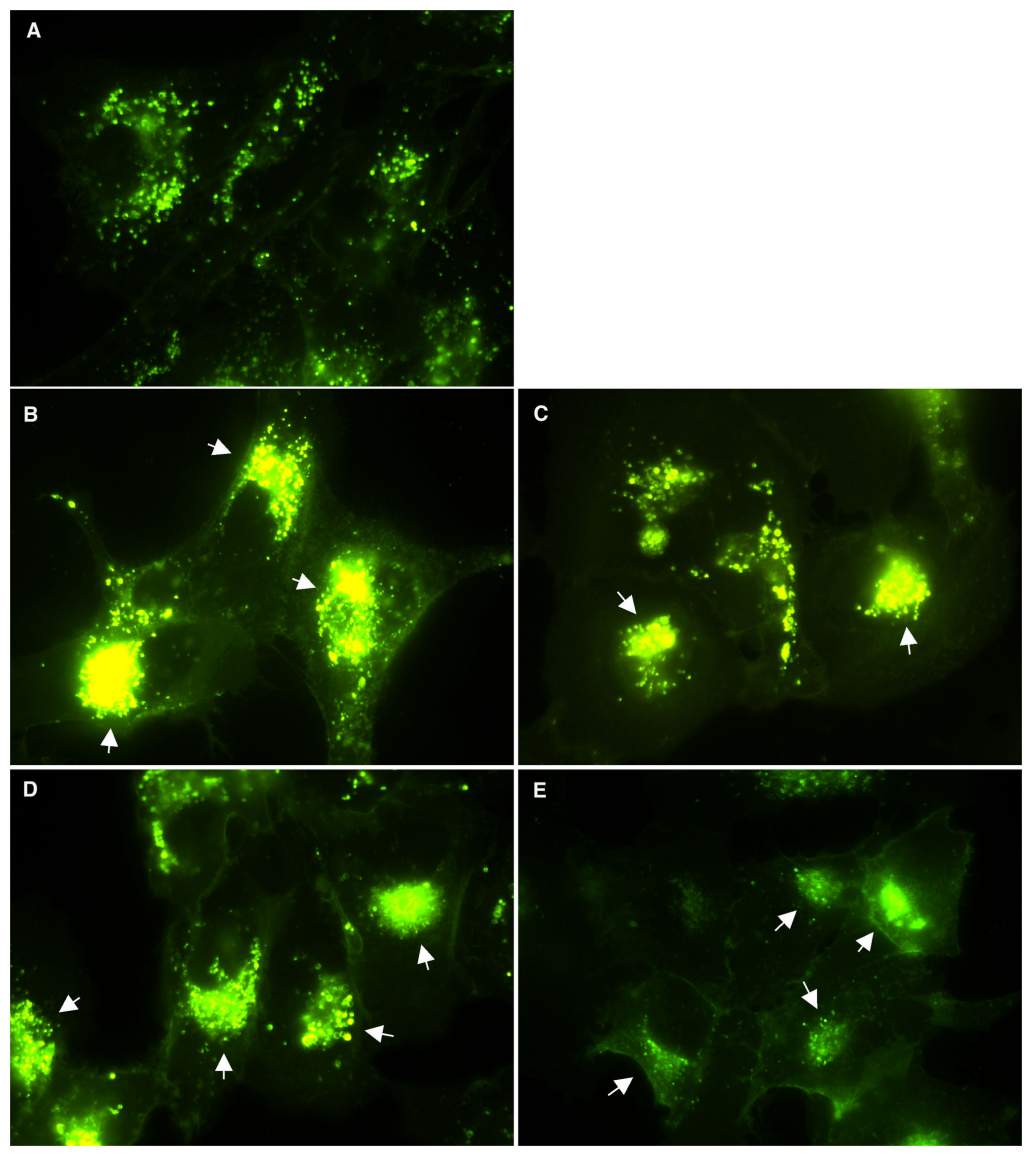
Effects of PKC activation on sphingolipid transport. Human NPC1 3123 cells were treated with (B) 20µM DCP-LA, (C) 2µg/ml oleic acid, (D) 2µg/ml linoleic acid, or 100nM PMA for 48 hours before BODIPY-LacCer staining was performed. In untreated cells (A), transport of the lipid to the TGN is inhibited and staining is visible only in punctate endocytic vesicles. In contrast, in treated cells the lipid can be seen in the TGN (arrows) in treated cells, indicating release of the transport block that characterizes NPC1.

To further confirm the positive effects of DCP-LA and fatty acids on the NPC1 phenotype, human NPC1 cells were treated with DCP-LA, fatty acids, or PMA to activate PKCs. Cells were then labeled with BODIPY-LacCer, which has previously been shown to provide a dynamic view of the endocytic pathway [30]. Following absorption to the plasma membrane, the LacCer sphingolipid enters normal cells via endocytosis and eventually reaches the trans-Golgi network (TGN; [30]). Due to the lipid transport block in NPC1 cells however, this sphingolipid is trapped in endosomes and its targeting to the TGN is dramatically inhibited [30]. Human NPC1 cells treated as indicated in Figure 7 show a dramatic improvement in lipid transport with the BODIPY-LacCer effectively reaching the TGN (Figure 7; arrows) compared to untreated cells that show only punctate, endosomal fluorescence (Figure 7A). These results provide further support that these agents are able to release the NPC1 lipid block.

Taken together, these results indicate that exposure to free fatty acids, which may act by activating PKC ε, has a positive effect on the NPC cholesterol storage phenotype and provide the rationale for further exploration.

## Discussion

We previously reported that Rab9 expression in NPC1 cells restored lipid transport from the E/L system and normalized cholesterol esterification [19] and subsequently showed that Rab9 was entrapped in insoluble vimentin filaments in NPC1 cells [5]. We hypothesized that accumulated lipids such as sphingosine [31,32] in NPC1 cells might exert an inhibitory effect on various PKC isoforms, resulting in a disruption of the vimentin phosphorylation/dephosphorylation cycle [19].

To characterize the nature of PKC inhibition and vimentin hyposphorylation in NPC1 cells, we expressed a number of PKC isoforms (α, βII and ε) in NPC1 cells and characterized their effect on vimentin solubilization and correction of the NPC1 phenotype. All three isoforms had a positive effect on vimentin solubilization to varying degrees (Figure 1). Furthermore, as we predicted, this PKC-induced vimentin solubilization was accompanied by the release of the entrapped Rab9 (Figure 1).

To further determine which PKC isoforms might be more effective in vimentin phosphorylation and release of Rab9, we tested eight different PKC isoforms in an *in vitro* assay. Interestingly, most isoforms were able to release Rab9 from vimentin to varying degrees (Figure 2), which may not be in case *in vivo*. This discrepancy is likely due to the different *in vivo* subcellular locations of PKC isoforms and their access to vimentin filaments [16,20]. However, it is also possible that vimentin may not be a direct substrate for certain PKCs, as has been shown with regards to PKC ε-controlled phosphorylation of vimentin [10]. In that study, PKC ε mediated vimentin phosphorylation, which was shown to be critical for proper integrin recycling through the cell. Our studies indicate that expression of PKC isoforms in NPC1 cells results in the partial correction of the NPC1 disease phenotype, i.e., cholesterol accumulation in the endosomal/lysosomal system.

Many studies have shown that long chain fatty acids such as oleic and linoleic, along with downstream metabolites such as DCP-LA, are able to activate PKC ε [24,25], an isoform shown to phosphorylate vimentin filaments [10]. Since we and others have previously reported that the availability of free fatty acids may be limited in NPC1 cells [27,33], we tested the hypothesis that exogenously added fatty acids might have a positive effect on the NPC1 phenotype, presumably by activating PKC ε and leading to phosphorylation of vimentin and release of Rab9. As predicted, addition of oleic acid, linoleic acid, or DCP-LA resulted in an increase of soluble vimentin in NPC1 cells (Figure 5). Furthermore, fatty acid addition resulted in a significant improvement in cholesterol esterification by NPC1 cells (Figure 3), indicating that lipid transport from the E/L system was restored. Finally, we tested the ability of diazoxide, a known activator of PKC ε [29], to correct the NPC1 phenotype, providing further support for the involvement of insufficient PKC phosphorylation of vimentin in contributing to NPC1 pathogenesis. In agreement with the results presented here, diazoxide was able to reduce cholesterol accumulation in NPC1 cells by 50% (Figure 6). Although our results with multiple PKC ε activators strongly suggest that PKC ε is mediating these changes within the NPC cells, we cannot exclude the possibility that these agents may be acting through some other pathway or protein besides PKC. Further studies to show PKC activation would clarify the role of PKC expression in the amelioration of the NPC1 phenotype.

Our data are consistent with previous observations of aberrant PKC expression in NPC mouse liver [34]. In those studies the expression of PKC α, δ, ε, and ζ were evaluated by immunoblot. Whereas PKC α and δ were about 3-fold higher in NPC1 livers compared to Wt livers, PKC ε was not significantly increased and PKC ζ was higher only in heterozygous livers. It is interesting to postulate that PKC ε does not render itself amenable to upregulation but can be activated, via fatty acids for example, and such activation can yield beneficial results in NPC1 cells. There is strong evidence that PKC ε is responsible for phosphorylating vimentin, which in turn controls the vesicular transport of various ligands such as integrins [10].

Considering the difficulties of delivering proteins as therapeutics, which are significantly amplified in diseases with neuropathology such as NPC1, a small lipid activator of a key regulator such as PKC ε would be greatly advantageous. These results suggest that identification of the PKC isoform(s) responsible for vimentin phosphorylation may provide new therapeutic targets for the treatment of Niemann-Pick type C disease and probably for a number of other lysosomal storage disorders with neuropathology that lead to E/L lipid accumulation [35].

## Materials and Methods

### Materials

Dulbecco’s Modified Eagle Medium (DMEM), trypsin, L-glutamine, gentamicin, and NuPage gels and buffers were obtained from Invitrogen (Carlsbad, CA) while FBS was from Hyclone, Thermo Scientific (Rockford, IL). The monoclonal anti-vimentin (V9), conjugated anti-mouse-IgG and anti-rabbit-IgG antibodies were from Santa Cruz Biotechnologies, Inc. (Santa Cruz, CA). The anti-GAPDH antibody was from Millipore (Billerica, MA) and the anti-Rab9 polyclonal antibody has been described elsewhere [27]. Filipin was from Polysciences, Inc. (Warrington, PA). Lumilight Plus substrate and FuGENE^TM^ 6 transfection reagent were both from Roche Diagnostics (Indianapolis, IN). [9,10-^3^H(N)] oleic acid (15 Ci/mmol) was obtained from NEN Life Science Products (Boston, MA) and LDL was from EMD Biosciences Inc. (La Jolla, CA). All other chemicals were acquired from Sigma-Aldrich (St. Louis, MO).

### Cell culture and transfection

The human wild-type fibroblast (GM05387), NPC1_0_ fibroblast (GM09341), and NPC1 fibroblast (GM03123) cell lines were obtained from Coriell Cell Repositories (Camden, NJ). The M12 Chinese hamster ovary (CHO) cell line and its wild-type parental line were a kind gift of Dr. Daniel Ory (Washington University, St. Louis, MO). Fibroblast cell lines were cultured in DMEM and CHO cells were cultured in DMEM/F12 (50:50) medium, supplemented with 10% FBS, 2 mM L-glutamine, and 50 µg/ml gentamicin in a humidified incubator at 37°C with 5% CO_2_.

The cDNA for PKC α (generous gift of Dr. A. Toker, University of California at San Diego, CA) was cloned into the bicistronic vector pIRES (Stratagene), which contains GFP for monitoring successful transfection. The cDNAs for PKC βII (generous gift of Dr. AC Newton, University of California at San Diego, CA), and PKC ε (ATCC) were cloned into vector pYDual, which expresses a nuclear-targeted RFP (Ioannou, unpublished). A Rab9-YFP fusion construct (described in [5]) was used for Rab9 expression. Transient overexpression was achieved by transfecting cells at 70% confluency using the FuGENE^TM^ 6 reagent (Roche Diagnostics) according to the manufacturer’s suggestions.

### Protein analyses

Transfected cells were harvested with PBS containing 2mM EDTA at 2 days post-transfection. Soluble and insoluble cell fractions were prepared as described previously [5]. Briefly, to obtain the soluble/cytoplasmic fraction, cells were incubated on ice for 30 min in cold “phospho” buffer [150 mM NaCl, 20 mM NaF, 100 µM Na _3_VO_4_, 20 mM Hepes, pH 7.5), 1% (v/v) Igepal, 10% (v/v) glycerol, and 1 µl/20mg tissue of protease inhibitor cocktail] and then centrifuged for 20 min at 14,000 rpm at 4°C; the clear supernatant was frozen in aliquots at a concentration of 1 µg/µl. The pellet (insoluble fraction) was washed 3 times in ice-cold PBS containing 2 mM EDTA and then resuspended in a volume of “Triton” buffer [PBS, 1% (w/v) SDS, and 0.1% (v/v) Triton X-100] equal to the “phospho” buffer. This solution was boiled for 10 min and sonicated until the solution became clear. The protein concentration of this fraction was adjusted to 1 µg/µl according to the protein concentrations determined for the soluble/cytoplasmic fraction. Protein concentrations were determined using the fluorescamine method as we have described [36].

For immunoblotting, 10 µg protein was resolved through a 4-12% Bis-Tris precast gel (Invitrogen, Carlsbad, CA) and then transferred onto a Protran membrane using an XCell II apparatus (Invitrogen, Carlsbad, CA) according to the manufacturer’s instructions. Blots were processed as described previously [27].

For Rab9 dissociation studies, 1.0 x 10^7^ NPC1 3123 cells were collected in ice-cold PBS and lysed by sonication 4 times for 10s each. The lysate was centrifuged at 14,000 rpm for 10 minutes to separate the soluble from the insoluble fractions and the total protein concentration was determined using a modified Bradford assay (Bio-Rad, Hercules, CA). An equal amount of each insoluble fraction was mixed with each purified PKC isoform from a PKC isozyme panel (Sigma, St. Louis, MO) and incubated for 60 min at 37°C. An equal volume of each sample was resolved through a 4-12% Bis-Tris precast gel, transferred to a membrane, and processed as described above.

### Cholesterol esterification

The preparation of [^3^H] oleate substrate and esterification assays were performed as previously described [5]. Cells were treated with 50µg/ml fatty acid for 2 days and then transfected with PKE α or PKC ε for 24 hrs before esterification assays. All values were generated in triplicate and normalized for total cell protein.

### Immunofluorescence microscopy

For filipin staining in transfected cells, cells were transfected with PKC or Rab9 using Fugene 6 according to the manufacturer’s recommendations. After 48 hrs, cells were stained with filipin as we have previously described [37]. Cells were mounted onto slides using Fluoromount-G (SouthernBiotech, Birmingham, AL) and analyzed on a Nikon Eclipse microscope fitted with a charge-coupled-device camera (Nikon, Melville, NY). Images were acquired with MetaVue software and then deconvoluted using AutoDeblur software from AutoQuant Imaging, Inc (Troy, NY).

For quantitation of filipin fluorescence, cells were seeded at 3 x 10^5^ cells/well in 6-well dishes and allowed to settle overnight, after which the medium was replaced with medium containing 10% lipoprotein deficient serum (LPDS) for 4 days. Cells were incubated with oleic/linoleic acids for 48 hours, DCPLA/DHA for 24 hrs, or diazoxide for 72 hrs before fixing and staining with filipin as we have previously described. Images were acquired using the same exposure time for all samples. Fluorescence intensity was determined using the integrated intensity function of MetaVue software; at least 150 cells were quantitated for each sample and each experiment was repeated 3 times.

For analysis of sphingolipid transport, cells were incubated with oleic/linoleic acids, DCP-LA, or PMA for 48 hours before BODIPY-LacCer staining was performed as previously described [30].
